# Biomimetic Functional Surfaces towards Bactericidal Soft Contact Lenses

**DOI:** 10.3390/mi11090835

**Published:** 2020-08-31

**Authors:** Tianyu Mao, Fengzhou Fang

**Affiliations:** 1 Centre of Micro/Nano Manufacturing Technology (MNMT-Dublin), University College Dublin, D04 V1W8 Dublin, Ireland; tianyu.mao@ucdconnect.ie; 2 State Key Laboratory of Precision Measuring Technology and Instruments, Centre of Micro/Nano Manufacturing Technology (MNMT), Tianjin University, Tianjin 300072, China

**Keywords:** biomimetic surface, functional feature, nano fabrication, contact lenses

## Abstract

The surface with high-aspect-ratio nanostructure is observed to possess the bactericidal properties, where the physical interaction between high-aspect-ratio nanostructure could exert sufficient pressure on the cell membrane eventually lead to cell lysis. Recent studies in the interaction mechanism and reverse engineering have transferred the bactericidal capability to artificial surface, but the biomimetic surfaces mimicking the topographical patterns on natural resources possess different geometrical parameters and surface properties. The review attempts to highlight the recent progress in bactericidal nanostructured surfaces to analyze the prominent influence factors and cell rupture mechanism. A holistic approach was utilized, integrating interaction mechanisms, material characterization, and fabrication techniques to establish inclusive insights into the topographical effect and mechano-bactericidal applications. The experimental work presented in the hydrogel material field provides support for the feasibility of potentially broadening applications in soft contact lenses.

## 1. Introduction

From the first introduction to microorganisms in 1674, the continuous observations and research in microorganism aggregation have contributed to the development of microbiology and pathology, which is an essential part of modern medical science. As a functional consortium of pathogenic microbe proliferation, biofilm plays a vital role in nutrient absorption from the outer environment and security protection for the constituted cell inside. Empirical therapeutic methods to deal with microbial infection are antibiotic utilization and surface chemical property modification, which may bring a toxic appendant effect to the in vivo organism. As manifested in the relevant literature, the progress rate of research in chemical therapeutics like antibiotics and topical drugs cannot keep up with the evolution of microbes and only have limited bactericidal efficacy in killing the top layer of biofilm, while the cell community beneath the outer surface retain high functionality and viability [1]. The bacteria protected in the biofilm generally develops resistance to survive in the specific antibiotic, and the stimulated multi-resistant pathogenic species could transfer the multidrug resistance to germs in community at high speed [2]. A 2019 Centers for Disease Control and Prevention (CDC) report concerning antibiotic resistance threats [2] confirmed that over 2.8 million antibiotic-resistant infections had occurred annually in the United States, and over 35,000 people had passed away as a result of these therapy-resistant diseases. Since clinical cases and theoretical analysis have shown the difficulty of coping with biofilm after complete microbial colonization, the precaution method before the initial stage of biofilm formation has become the leading direction for biofilm development mitigation. According to the previous review on bacterial adhesion [3], to the detriment or prevention of biofilm formation, the initial attachment of a single cell is not only the first, but also the most susceptible stage in the biofilm formation process.

For the selection process in nature, it is a challenge for flora and fauna to tackle biofilm formation and ramification. Hence, the epidermal topography of this biology has evolved to generate unique surface appendages, for instance, insect wings (cicada, dragonfly, etc.), marine animal skin (shark etc.), and plant leaves (taro, lotus, etc.). Based on the investigation of the antibacterial properties of these natural occurring antimicrobial surfaces [4,5,6,7,8,9,10,11,12,13], the antibacterial activities could be divided into two categories: inhibition activities and biocidal activities. Inhibition activity is utilizing the low-surface-energy approach to realize bacterial adhesion prevention [14], while biocidal activity kills the germ once initial bacterial adhesion has taken place. The results of analysis of these investigations revealed that the surface topography is the predominant factor to determine the surface functionality of which microstructure from marine animal skin and plant leaves imparts the inhibition property to the cell–substrata interaction interface and the nanostructure from insect wings possesses a biocidal property lethal to pathogens.

As the validity of the topographical function of natural occurring antimicrobial surfaces has been confirmed by various cell incubation tests and theoretical model simulations, several biomimetic surfaces have been fabricated with surface modifications referring to antetype surface geometrical features, which has prompted the feasibility analysis of these topographical effect applications in the biomedical field. The subsequent comparative analysis of biomimetic surfaces and antetype basis has demonstrated that biomimetic surfaces possess higher antibacterial capabilities by controlling the geometrical parameters and spatial distribution of the nanostructure, so the phenomena illuminated that surface bactericidal efficacy and other surface properties could be modified by varying the surface features.

Contact lens related keratitis is commonly induced by continuous pathogen adhesion from various exteriors, where the keratitis is corneal inflammation that threatens serious complications with permanent damage to vision performance and even blindness. Disposable contact lenses, especially daily disposable contact lenses, have adopted a minimal lens care design strategy that can significantly reduce care and maintenance procedures requiring complete sterile conditions. However, high risk for bacterial adhesion and inducing microbial keratitis is reserved in ubiquitous extended-wear or continuous-wear behaviors. Considering the extensive application and functionality of biomimetic surfaces, the introduction of an antibacterial nanostructure to the soft contact lens (SCL) edge area could be a potential solution to extend the expiration date of SCL and maintain a high level of hygiene when encountering a complicated service environment.

## 2. Bacterial Adhesion and Rupture Mechanism

### 2.1. Bacterial Classification and Membrane Structure

**Figure 1:**
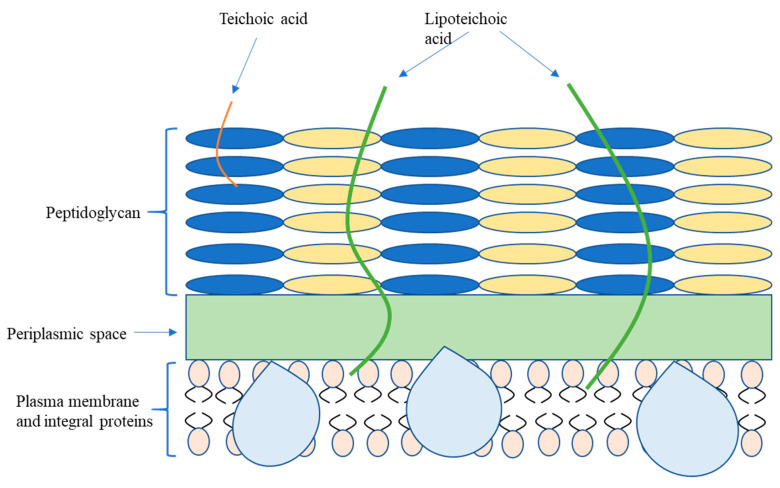
Cell structure of Gram-positive bacteria.

**Figure 2:**
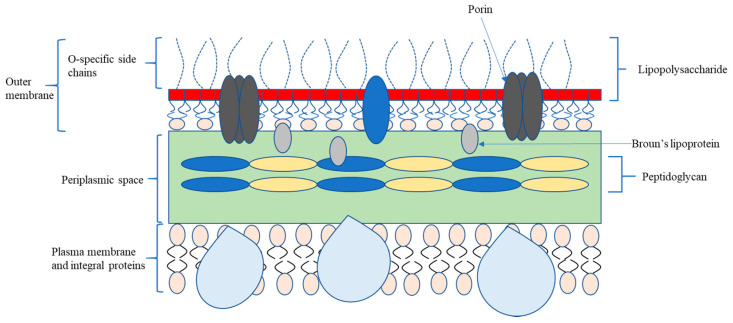
Cell structure of Gram-negative bacteria.

Bacteria are typically classified referring to morphology (spherical-cocci, rods-bacilli, and helical-spirochetes) and cell wall (Gram-positive and Gram-negative bacteria) [15]. The figures below depict detailed schematics of two different cell wall structures that consist of Gram-negative and Gram-positive bacteria, where there are several regular structural compositions in both types of cell membranes. Distinctive structural compositions like organelles could respectively determine unique competence in repelling toxicity and mechanical stress from the outer environment. The surface properties may vary in the aspect of age and medium composition, while some instinctive biological characteristics of cell envelopes remain constant. As shown in Figure 1, the thick cell outermost layer of the Gram-positive bacterium is constituted by cross-linked peptidoglycan, teichoic acid, and lipoteichoic acid; one of the Gram-negative bacteria is a compound layer comprised of the peptidoglycan layer and phospholipids and lipopolysaccharides (LPS), which is presented in Figure 2. In the Gram-staining procedure, Gram-positive cells retain a violet dye while Gram-negative cells fail to retain the violet dye due to a thinner peptidoglycan layer [16]. As the first barrier connecting to the periplasm and outer environment, the outer membrane has the capacity to maintain structural integrity and toxicity recognition. The outermost layer for Gram-positive bacteria that lacks the corresponding outer membrane in Gram-negative bacteria is inversely thicker than the one of Gram-negative bacteria. The thicknesses of the peptidoglycan layers for Gram-positive and Gram-negative bacteria are 20–40 nm and 5–10 nm, respectively. The osmotic pressure inside the Gram-positive bacteria is equivalent to approximately 20–25 atmospheres, so the resilient and enduring membrane is able to withstand the high internal turgor pressure of Gram-positive cells without rapid deformation. The low internal osmolarity in Gram-negative bacteria that induces thin peptidoglycan also determines the compressive capacity of cell outer layer [17]. Despite the high mechanical strength, peptidoglycan synthesis could be selectively blocked by antibiotics, which results in cell lysis. Current antibiotics have been developed to have high selectively toxicity [18] to target pathogenic germs without mortal negative impact on ordinary cells. Due to the embedded discriminative porins on the outer membrane, current chemotherapeutic agents are deficient in penetrating into Gram-negative bacteria [19]. Along with low permeability from predators, mutation hidden beneath the biofilm could promote antibiotic resistance inside the biofilm community via acquisition of foreign DNA through horizontal gene transfer, which benefits from a unique genetic plastic [20].

Most peptidoglycan layers targeting antimicrobial agents primarily maintain sustainable clinical efficacy in Gram-positive bacterial infection, but some Gram-positive bacteria like Methicillin-resistance S. aureus (MRSA) are recently observed to possess increasing resistance rates.

### 2.2. Pathogenic Microorganism on Various Exterior

Microbes are ubiquitous in every corner of living quarters, and high adaptability to hostile environment increases their survivability. Parasite pathogens inhabiting plants and livestock could disperse through the airflow, and airborne bacteria could reach the normal human range of activities in an indoor environment. The major portion of bacteria may not develop a large community and low microbial concentration would have a confined impact on human health, but the nosocomial infection or neonatal infection occurring in hospitals could always lead to lethal risk which is shown in Table 1. Research concerning catheter-associated urinary tract infections or ventilator-associated pneumonia has generally focused attention on invasive medical device associated infections, where the accepted explanation for these infections is that in vivo organisms are exposed to pathogens by invasive device utilization.

Like parasitic organisms, pathogens live and procreate based on nutrient absorption from the host organism, so microbial colonization or parasitism could have occurred in every lifeform on Earth. As the human body is commonly retained at constant temperature and continuous metabolism, microorganisms have evolved specific mechanisms to maintain sustainable parasitism activities in this desirable breeding ground [15]. In a complex ecosystem, 10^13^ mammalian cells and approximately 10^14^ bacteria, fungal, and protozoan cells generate a complicated symbiosis relationship [21]. These microbes are distributed in different parts of in vivo organisms including the otolaryngology system, urinary system, and viscera system. Distinct from the common impression on microbes, normal microbial inhabitants in the human body occasionally make disturbances if immune systems become debilitated or the microbes gain access to the host tissue without correlative biocide agents. Pathogens produce highly specialized effectors for evading host immune responses and modulating host cell survival on order to develop co-evolution and a symbiotic relationship with hosts [22].

As a majority of bacteria encountered in daily life are innoxious, common bacteria could autonomously execute most of the basic metabolic activities, as the primary reason for inhabiting the host is seeking rich nutrition [15]. Among the different parasitism forms and predisposing mechanisms of pathogens, pathogens can be divided into obligate pathogens and facultative pathogens. Obligate pathogens rely on the cellular machinery of host cells and require multiple hosts in different life stages [23]; all viruses are obligate pathogens. Most facultative pathogens are environmental bacteria that only cause disease when encountering a susceptible host [24], while opportunistic pathogens are normally commensal, but accidently spread disease to the injured or immunocompromised host [25].

Nosocomial infection mainly manifests as a clinical disease instead of microbial colonization because most of the predisposing bacteria for nosocomial are opportunistic pathogens. Based on previous study [26], *Escherichia coli* and *Staphylococcus aureus* are the conventional isolated nosocomial pathogens [27]. Since *E. coli* is detected in a large proportion of urinary tract infection (UTI) clinically collected specimens from female patients, it rarely exists at other infection sites [28]. Therefore, precautionary measures in sterilization during operative treatment and postoperative care could effectively minimize the risk for major nosocomial infection.

### 2.3. Bacterial Colonization and Biofilm Formation

Microorganisms have the propensity to crowd together for biofilm formation on biotic or abiotic surfaces, which is a heterogeneous and multifunctional community that maintains firm adhesion to the befitting substratum. This assembled lifestyle presents numerous advantages compared to free-swarming planktonic cells including increased nutrient availability and higher security from predators [29]. Biofilms are destructive to human health and industry: as a functional aggregation of microbes, biofilms are accommodated at surfaces as diverse as the natural liquid–gas interface, water purification system and corners for industrial equipment, and indwelling medical devices in vivo [30]. The motility and micro-scale volume of pathogens increases the difficulty for the antibiotic to reach and eradicate the microcolonies hiding deeply in the host tissue with poor drug permeability. On the other hand, biofilms can be used for technical innovation with exclusive biotechnological processes [31] including bioremediation [32], waste purification [33], biological fuel cells [34], or the production of biofertilizers [35].

**Figure 3:**
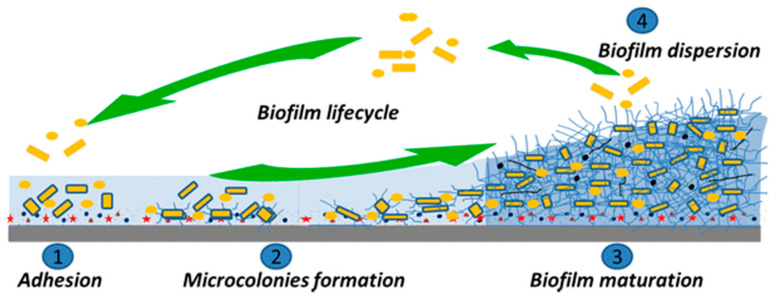
Schematic of biofilm formation in different stages [36].

Bacterial adhesion is the prerequisite for biofilm formation; in the process from planktonic to the sessile state, the transition process contains two different stages that are termed as reversible adhesion and irreversible adhesion. Reversible adhesion means that the cell–substrate bonding is weak and cells could return to the aqueous medium by small external force; irreversible adhesion is initiated by multiple contacts between the cell and substrate [29]. After irreversibly attaching on the nutritional host, multicellular microcolonies are formed by exponential cell division and develop for biofilm maturation. When receiving the signal from sufficient conditions in an external environment or deficient conditions in an internal environment condition, for instance, limited nutrient accessibility or restricted oxygen levels, dispersed cells would be activated to be released to the outer environment by the maturated biofilm. Dispersed cells would repeat the growth process and attach to a new accommodating surface to initiate the next colonization. In conclusion, the biofilm development lifecycle can be divided into four stages in Figure 3: (1) initial attachment of microbes to a substratum or each other; (2) formation of microcolonies; (3) biofilm maturation; and (4) biofilm dispersion [36]. After irreversibly attaching to a substrate and being embedded in a matrix of extracellular polymeric substances (EPSs) that they produce [37], later activities of microbe proliferation would be sheltered by the mature biofilm. Therefore, the initial attachment of single cells switching from planktonic status to the surface is a critical stage in the process of biofilm formation.

Aside from the benefits from biofilm formation, the strengthened adhesion on the substratum has several lethal limitations including the inhibition of motility. When the nutrition source is depleted, the reinforced bond would become a barrier for detained bacteria with a shortage dilemma. In the conversion process from the planktonic to coaggregation state, the transcriptional regulator inversely controls the production of motility and EPS, which means that the required motility for escape switches to EPS secretion [38].

### 2.4. Interaction between Bacteria and Topographical Surface

#### 2.4.1. Conventional Bacterial Adhesion Mechanism

The biosorption mechanism is critical in considering an antifouling strategy design that elucidates the cell–substrate interaction and its influence factors. The connect strength between cell and substrate depends on the microbes type, substrate condition, and surrounding environment [39]. As initial attachment is manipulated by multi-stage physical adhesion force, fluid mechanics from the outer environment combined with self-motility from extracellular appendages like pili and fimbriae are the major driving force approaching the near-surface position. Hydrodynamic forces and electrostatic forces are dependent on the surface wettability and charge [40], and drive force like van der Waals forces need to overcome the barrier, like an electrostatic repulsive force to deliver the bacteria to their destination. To maintain intimate contact, EPS, which exhibits both viscous and elastic properties, is secreted in the stage of irreversible adhesion as the adhesive force in the irreversible adhesion stage can anchor the cell to the substrate steadily, which confines the motion range of bacteria [41].

Suspended in a bulk liquid environment, bacterial transport to solid is likely dependent on the Brownian motion in sedimentation and convective diffusion process [42]. Hydrodynamic force and electrostatic force are vital influence factors for bacterial passive movement; surfaces with notable hydrophilicity or hydrophobicity significantly reduce the level of cell adhesion [43] as hydrophobic surfaces reduce the repulsive force between the cell and substrate [44], which exhibits higher attraction than the hydrophilic surfaces [45]. When the cell membrane is hydrophobic, it will partially adhere to hydrophobic materials and vice versa. Thermodynamic theory indicates that the surface energy of the cell determines the preference of adhering substrate, where cells with a larger surface energy than the suspended liquid attach to the hydrophilic surface, which possesses a large surface energy, so the common cell with a lower surface energy than the suspended liquid environment prefers to attach to a hydrophobic material that has a low surface energy [46]. Similar to the hydrodynamic condition, the cell membrane is commonly negatively charged, and has a preference to attach on a substrate with a positive to neutral charge [47]. When it achieves proximity to the near-surface position, thermodynamic and Derjaguin–Landau–Verwey–Overbeek (DLVO) could explain the initial attachment by postulating that the bacterial surface is smooth without the consideration of bacterial appendages inducing membrane surface roughness.

Electrostatic interaction involves ionic strength and pH of the surrounding liquid medium; low ionic strength could increase the energy barrier to prevent the approaching bacterial from Brownian motion or self-motility [48]. Van der Waals forces are more predominant in the vicinity region of the substrate [49], the force would decrease dramatically in increasing separation distance despite the long application range. For a short range within 1 nm distance to the substrate, acid–base interaction forces become dominant, while it decreases exponentially as the separation distance increases [50]. Concerning the cell wall deformation in the process of bacterial adhesion, long range forces like van der Waals forces could be significantly affected by the slight membrane deformation [51].

#### 2.4.2. Perspectives of Contemporary Nanostructure–Membrane Interaction

For the substratum property, there have been abundant explorations on the impact on cell adhesion by surface chemical property modification, but the recent trend has turned to focus on the investigation of the surface topographical effect. The major reason for this shift is from the comparative analysis of cell adhesion on different surfaces with identical surface structure and distinct surface chemical characteristics, where the results revealed a narrow difference existing between the wo samples, which emphasizes the dominant influence of surface topography on antibacterial property [13].

There are currently two main antibacterial strategies including the inhibition mechanism and detriment mechanism. The inhibition mechanism repels bacterial adhesion based on the low surface energy approach and the detriment mechanism employs a nanostructure to create an unfavorable contact interface and impairs the cell membrane structure, which causes membrane rupture and cell death.

In the early stage of research into the topographical effect, some natural innate antibacterial surfaces from the flora and fauna have generally attracted mainstream attention like the lotus and shark skin. The main difference between the smooth surface with this antifouling surface is the high-aspect-ratio structure, which bestows the hydrophobic feature and self-cleaning ability. The geometrical features of the lotus or shark skin are superhydrophobic, which was nominated as the foremost factor in antibiofouling activities as a superhydrophobic surface combined with a high-aspect-ratio structure induces a low surface energy phenotype [52]. A low surface energy polymer formed into a smooth coating ($R_{a}<6nm$) by Tsibouklis et al. has been continuously monitored as evidence to prove the feasibility for inhibiting the bacterial adhesion mechanism [14].

Unlike the inhibition mechanism, the contact-killing mechanism prevails in insect wings with a high degree of hydrophobicity like the lotus and shark skin. When investigating the antibiofouling property of the cicada wing surface, Ivanova et al. [13] found that the hydrophobicity of the surface had a limited effect on the bacterial adhesion, and the bacteria was found to have inactivation on the incubation surface instead of the preconceived repellent phenomena. With the intention to determine the correlation between the surface chemistry of the cicada wing and exhibited lethality to *P. aeruginosa*, magnetron sputtering was employed to cover the surface of the cicada wings with a 10 nm-thick gold film. Some surface characteristics like wettability were modified by the gold coating, and water contact angle substantially decreased from 158.8° to 105.5°. Results of comparative AFM roughness analysis in the processed specimen and control specimen confirmed that the surface morphology profiles of the two samples were virtually identical. The bactericidal effect of the gold-coated wing was preserved, which proved that the topographical effect of the nanostructured surface was essentially responsible for the lethality of the cicada wing instead of the surface chemistry [13]. Based on the bactericidal model found on cicada wings, Pogodin et al. [53] proposed a biophysical model for interpreting cell–nanostructure interaction. The biophysical model assumes the cell membrane as a thin elastic layer without appendages by employing numerical surface free energy model and suggests that the membrane rupture point occurs in the region suspended between the nanopillars. The premise of the cell rupture model is sufficient adhesion strength to restrict the cell movement, or else, the mobile cell could leave the unfavorable environment once receiving the response signal. To promote neuron-to-electrode interface activities, Xie et al. [54] reported that the utilization of nanopillar arrays to pin the position of neurons would enhance the cell attachment more than a flat substratum. The exhibiting hysteresis movement on the nanopillar pattern could explain the firm binding force between the nanostructure and cell. Xue et al. [55] constructed a mathematical model to illustrate the theoretical mechano-bactericidal mechanism of the nanopatterned structure and the stretching theory designating gravitational force combining nonspecific forces like van der Waals force as the drive force for membrane deformation. Li et al. [56] created a quantitative thermodynamic model by means of surface free energy analysis, and the model elucidated that more cell-substrate contact areas was the key parameter in the improvement in topographical effect. The contact area could be expanded via surface roughness adjustment through nanostructure spatial dimension amendment, which could consequently enhance the bactericidal capacity. Furthermore, the model indicated that high-motile bacteria are less resistant to nanopatterned surfaces [57]. Liu et al. [58] implemented an investigation into the interfacial energy gradient between the cells and nanopillars, which was proposed as the driving force to promote cell adhesion, and the results implied that nanopillar parameters were a substantial influence factor of the interfacial energy gradient. Higher aspect ratio could exert greater pressure on the cell membrane, while smaller cell volume would be exerted at higher pressure with larger contact angle.

Bandara et al. [8] first introduced EPS to explain the bactericidal mechanism of nanopillars, where the model of the adhering cell wall was covered by EPS instead of a simple thin layer directly in contact with the nanopillar. The bacterial damage was extrapolated as being initiated by the coefficient from adhesion and shear force, and membrane damage may occur from the top side of the bacteria instead of the contact bottom side due to turgor pressure against the peptidoglycan (PG) layer damage. The durability of the nanostructure could be affected by the weak modulus of the nanopillars, and the scanning electron microscope (SEM) image of the results showed the bending nanopillar. Based on the flexible property of nanopillars, Ivanova et al. [59] demonstrated that the deformation of nanopillars could be inverted into mechanical energy storage that would impose lateral stretching to the cell membrane. When characterizing the physiological and morphological effect of titanium nanopillars on bacteria, the nanopillars induced the membrane deformation and penetration instead of rupture or lysis. The nanopillar was found to constrain the cell division and provoke the synthetic of reactive oxygen species, which indicated the antibacterial property of the nanostructure could be mediated by oxidative stress [60].

## 3. Bactericidal Properties of Natural Antimicrobial Surfaces

### 3.1. Naturally Occurring Antibiofouling Surfaces

Current research into natural innate antifouling surfaces provide insights into the topographical effect and cell–substrate interaction mechanisms, which establishes avenues for reverse engineering to fabricate synthetic analogues.

Instead of relatively sterilized living conditions in modern human society, dust particles and microbes are ubiquitous in the natural environment and are actively seeking opportunities to colonize and proliferate. In the process of natural selection, the retentates generally develop various strategies to cope with microbial contamination. For an initial understanding of the natural antibiofouling property, inchoate studies have focused on the relationship between non-stickiness phenomena and hydrophobicity. Hydrophobicity, as a property of expelling water, widely exists in plant leaves and insect exterior surfaces. Based on the observation of antifouling activities on hydrophobic surfaces, hydrophobicity was presumed as the primary factor of self-cleaning activities that inhibit cell adhesion.

Natural flora like taro and lotus have amassed a delicate array of structures that could provide effective protection from microbial colonization. Ma et al. [5] first investigated the physicochemical interaction between topographic structure on taro leaves and colloidal particle or bacteria under submerged conditions, where the results demonstrated that nanoscale surface features could be attributed to the resistance to both biological and nonbiological particles under the partially or completely wetted condition. The research revealed that the resistance to bacteria or abiotic particles for lotus leaf would remain consistent regardless of the wetted or nonwetted conditions, and that dense nanosized surface features on the leaf could be the source of bionic engineering to control foulant particle adhesion. Barthlott et al. [61] conducted a comprehensive review of the lotus effect containing low drag and antibiofouling characteristics and its bionic surface technologies, illustrating that hierarchical microstructure morphology on the leaves’ epithelium is a prerequisite for its superhydrophobicity characteristic. Aside from the plant leaf, shark skin is also a distinguished mimic prototype of numerous biomimetic surfaces that have been engaged in numerous commercial applications. Chien et al. [6] described the relationship between bacterial initial attachment interference and microstructure on shark skin and the influence factors of surface roughness and wettability. Although the enhanced initial attachment could be attributed to surface roughness in some cases, high roughness eventually played a central role in inhibiting biofilm formation. Lower amounts of biofilm appeared on these patterned surfaces compared to flat surfaces, and the composed protrusions of microstructure was deduced to hinder microcolony expansion and interfere with bacterial quorum sensing.

Like marine and botanical biology, which possess the motility requirement in their natural inhabited environment, many insects have the inclination to minimize foreign particle adsorption for the purpose of maintaining functionality [13]. The dust or dirt particles diffused in the air, or airborne bacteria may adsorb to the insect for either nutrition absorbing or utilizing insect mobility. To avoid the complication from bacterial adhesion, the outer layers of insects’ wing surfaces have topographically evolved to possess an extreme degree of hydrophobicity, which is designated as low adhesiveness by limiting bacteria anchoring on the surface. However, in the research into interaction between insect wings and bacteria, the hydrophobic characteristic could not provide prolonged resistance to bacterial adhesion [13]. Ivanova et al. [13] identified no direct connection between hydrophobicity induced self-cleaning activities and antibiofouling property, and the antimicrobial property of the surface of cicada (*Psaltoda claripennis*) wings were discovered to be a contact-killing mechanism instead of a bacteriostatic approach accepted by plant and marine biology. In the supplementary test [12] against several bacterial species with wide range cell morphology and wall type, the topographical effect of wings were observed to consistently kill Gram-negative cells, while the Gram-positive cells that incubated on the wings remained intact. The morphology of the cell wall was verified to have a limited impact on cell susceptibility to nanostructure. After the contact-killing mechanism was validated, the direction of mechano-bactericidal research turned to find natural bactericidal surfaces detrimental to extensive Gram-positive and Gram-negative cells.

To identify versatile natural bactericidal surfaces and investigate the potential for bactericidal surfaces incorporating natural biocidal nanopatterns in a biomimicry design, Ivanova et al. [62] mimicked the high-aspect-ratio nanopillar on dragonfly wings and transferred the structure to a silicon wafer. The native and mimetic surface were both assessed to possess high bactericidal efficacy to both Gram-negative, Gram-positive bacteria, and even the multi-layer spore coat. The experimental results not only confirmed the existence of optimal antimicrobial surfaces, but also determined the feasibility of transferring the antibacterial property to biomimicry surfaces.

### 3.2. Bactericidal Efficacy of Natural Nanostructure Surface

As shown in the Table 2, cicada (*P. claripennis*) specimens were collected for the validation test for both the Ivanova et al. and Hasan et al. [12,13] models, and the nanopillars on the specimens’ wings were 200 nm tall, 100 nm, and 60 nm in diameter at the base and cap, respectively, and 170 nm was the interpillar distance. The values of cell membrane deformation, for instance, cell sinking distance and rupture points, were recorded by atomic force microscopy (AFM) with the tip positioned on the top of a single cell attached to the wings’ surface with constant force. The rupture point occurred when the AFM tip measuring the cell downward movement lowered approximately 200 nm over about 220 s before a sharp drop. The bacterial cultivation experiment not only revealed the bacterial susceptibility to nanostructured wing surfaces rather than surface chemistry, but also clarified stereotypical theory concerning the superhydrophobic characteristic and antibiofouling property.

Hasan et al. [12] implemented a complementary investigation to the range of cell lethality and relevant bactericidal efficiency for cicada wings by increasing the test species from one kind of Gram-negative bacteria to four Gram-negative bacteria (*B. catarrhalis*, *E. coli*, *P. aeruginosa*, and *P. fluorescens*) combining three Gram-positive bacteria (*B. subtilis*, *P. maritimus*, and *S. aureus*). The results from the cell incubation tests showed the consistent lethality to Gram-negative cells for cicada wings regardless of cell morphology (coccoid-shaped or rod-shaped), but Gram-positive cells remained viable and functional in comparison with cell attached to the glass control surface.

Kyle et al. [9] selected several usual insects (cicadas and sanddragon) against eukaryotic microorganisms (*S. cerevisiae*–yeast cell) to explore the prominent factors for bactericidal effect and cell rupture mechanism. Three species had different aspect-ratios (0.5, 1.8, and 4.6) and water contact angle (hydrophobicity and hydrophilicity), and a comparison of viability loss of yeast cell on different surfaces proved that a nanostructure with a higher aspect-ratio would perform better in cell rupture activities. Resultant analysis suggested that adhesion strength between the cell and nanostructured surface is a critical impact factor in the microbial mechano-rupture process, and the high-aspect-ratio nanostructure possessed better cell affinity than one with low-aspect-ratio. Durability of the nanostructure was also first prompted in the observation of nanostructure damage by direct interaction between yeast cells and nanostructure, where nanostructure removal also provided proof of intimate adhesion between yeast cell and nanostructure.

To elucidate the relationship between the nanoscale topography and the degree of bactericidal activities, Kelleher et al. [7] carried out a detailed investigation on the topography characterization of three specimens of cicada wings by SEM and AFM. The measured wettability and surface energy illustrated the close connection between the nanostructure geometrical parameters and hydrophobic characteristics and low surface energy.

Like the cicada, the dragonfly also has a high-aspect-ratio nanoprotrusion structure on the wings’ epicuticle lipids. David et al. [63] selected three species of dragonflies from a similar inhabited environment to explore the bactericidal capabilities against various microorganism types including Gram-negative bacteria, Gram-positive bacteria, and spores. Unlike cicada wings, wings of all species were lethal to all bacterial types, which provided a template of hybrid functional surfaces that possess resistance to all kinds of bacterial contamination.

Instead of targeting insect cuticle structure, the work of Li et al. [64] focused on the antibacterial properties of gecko skin nanotipped spinules and its equivalent acrylic replica to tackle Gram-positive (*S. mutans*) and Gram-negative (*P. gingivalis*) bacteria. The recorded data showed that both non-viable *S. mutans* and *P. gingivalis* bacteria had a significant rise (66% and 88%, respectively) on gecko skin topography after seven days within a promotive liquid environment, and the validated promising antibacterial character of plastic resin replica design offers a potential anti-biofilm coating in biomedicine and biomedical instruments.

Although the proposed model from Kyle et al. [9] revealed the risk for nanostructure durability, Bandara et al. [8] first introduced the mutual damage behaviors caused by immobility bacterial movement in the research of interaction between nanotextured surfaces (NTSs) on dragonfly wings and Gram-negative bacteria (*E. coli*). The model depicting the cell–nanostructure interaction mechanism generated in the research showed that the membrane rupture was due to strong binding adhesion between NTSs and bacteria via secreted EPS and shear force exerted by nanopillars when the bacteria attempted to get away from the biocidal surface.

Through the awareness of a lack of information concerning bacterial growth stage, Truong et al. [65] prepared *P. aeruginosa* and *S. aureus* bacteria at different physiological growth phases to evaluate whether the growth phase of the tested cells had any impact on the cell susceptibility to the nanostructured surface. The selected natural antimicrobial surfaces were collected from damselfly specimens, and selected bacterial cells from different growth phases were cultivated on the wing surfaces for 3 h. It was found that both bacteria were highly vulnerable to mechanical rupture by the damselfly wing surfaces at the early (after 1 h and 6 h of growth) and late (after 24 h) stationary phase, and immature *S. aureus* and mature *P. aeruginosa* had a greater tendency to attach than those of the bacteria in the aged phase.

As the high bactericidal effect of dragonfly wings has been demonstrated in previous studies, giant unilamellar vesicles (GUVs) were designated by Cheeseman et al. [10] to probe the fundamental interaction between two dragonfly specimens. GUVs are an adjustable synthetic membrane model system with precise composition, which is suitable for variation control analysis, and alternative features include membrane composition, inner turgor pressure, and morphology size [56,57]. Some pieces of native wing surfaces were covered by gold film, and the membrane rupture of GUVs occurred with cell adsorption on the substratum in both the native state and gold-coated state. The collected experimental data first demonstrated that the tension force involved in the membrane–nanostructure interaction was in excess of 6.8 mN m^−1^.

## 4. Artificial Biomimetic Surface Development

### 4.1. Characterization of Bio-Inspired Surfaces

Reverse engineering has contributed to the development of the biomimicry field by mimicking natural resources. In the process of investigating the relationship between antimicrobial property and topographical effect, in Table 3, some attempts to mimic the geometrical features of naturally occurring antibacterial surfaces have been implemented. After first discovering the bactericidal property of the cicada wing, Ivanova et al. [62] developed a hydrophilic nanomaterial named as black silicon (bSi), where the reactive-ion etching (RIE) technique was utilized to fabricate high-aspect-ratio nanoprotrusions based on the natural antetype dragonfly *Diplacodes bipunctata*. The samples of the cell incubation test on the bSi surface contained Gram-negative bacteria, Gram-positive bacteria, and endospores, and the experimental results demonstrated a high bactericidal efficacy (average killing rates of up to ~450,000 cells min^−1^cm^−2^). A multilayer spore coat was also observed disrupting, so the feasibility model of the topographical effect applied in tackling bacterial adhesion was validated.

Inspired by the bactericidal model from bSi, Hasan et al. [66] prepared a hydrophobic silicon nanostructure with modified geometrical features through deep reactive-ion etching (DRIE). Compared to the bSi surface, the most remarkable difference in the hydrophobic silicon nanostructure was the height, which possessed a pillar height almost eight times that than bSi. In the research not only involving Gram-negative and Gram-positive bacteria, the mammalian cell (mouse osteoblast) was also taken into consideration to analyze the potential risk for human cell lysis caused by interaction between the nanostructure and in vivo organism. The cell validity incubated on the processed surface was close to six-fold lower than on the control sample (unmodified silicon wafer), so the fabricated nanostructure would lead to substantial detriment to all tested cells including the mammalian cell. The feasibility of nanostructure applications in prothesis or other medical implants extends to surgical instruments, which have intimate contact with mammalian cells in vivo, to maintain ultraclean and aseptic conditions requires further systematic investigation.

As a promising polymer material, polymethylmethacrylate (PMMA) is widely applied in the construction of medical devices such as microsensors, bone cement, and drug delivery applications due to its biocompatibility and low risk for foreign body reaction. Mary et al. [67] used soft lithography to fabricate a bactericidal physical surface topography inspired by the nanostructure on a cicada wing on the PMMA film to evaluate the predominant factors for bactericidal efficiency against Gram-negative bacteria (*E. coli*). Compared to flat films, the nanostructured surface had a lower density (67–91% of densities on flat films) of adhering bacteria, and a higher number of non-viable bacteria (16–141%) than the flat films. In the discussion on the major influence factors of bactericidal efficiency, the minimum threshold for the optimal interpillar distance was found to be between 130 and 380 nm through the quantitative analysis of cell orientation data, where closer spaced nanopillars performed better in antibacterial activities.

Ito et al. [68] proposed metal addicted assisted etching to mimic a cicada wing surface on a silicon substratum that was also capable of controlling the dimension flexibly, where the fabrication method mainly used was the wet process, which is mature precision technology widely used in large area fabrication. The antibacterial property of the fabricated structure was confirmed in the evaluation process where the concentration of viable bacteria on the fabricated structure decreased to lower than 1 CFU/mL after the 24 h test.

Bacterial deposition on optical devices like endoscopic devices, microscopic slides, and contact lenses has brought extensive concern in the hygiene problems of biomedical optical devices. Han et al. [69] presented the first hydrophilic transparent nanopillar-structured surface with bactericidal property on a quartz substrate via nanosphere lithography, and the anti-fogging and anti-reflective properties were also probed in the research. Two typical Gram-negative bacteria (*P. aeruginosa* and *E. coli*) associated with various eye diseases were selectively incubated on the fabricated nanopillars, and the highest bactericidal efficiency was observed in the test on nanopillars that were 300 nm in height and 10 nm in apex diameter. Furthermore, the properties including superhydrophilic, anti-fogging, and anti-reflective were confirmed by water contact angle and light reflectance measurement, which proposed a potential hybrid functional optical device.

Despite lacking previous study on the bactericidal activity of moth-eye surface, several unrelated species with similar nanocone features shared the same antibacterial mode-of-action, which proposes a tendency of convergent evolution. Therefore, Felipe et al. [70] designed a mimicking moth-eye nanostructure on a flat PMMA thin film to achieve antibacterial functionalities through thermal polymer nanoimprint. Aside from typical pathogenic bacteria, the cytocompatibility of the topography toward human keratinocytes (HaCaT) was also explored. Keratinocytes were selected because of their vital role in the outermost layer construction of skin and epidermis regeneration [71] for patients with wounds. Due to the hydrophobicity of the mimetic surface, the relationship between surface wettability and cell adhesion was determined by bacterial cultivation. The topography turned completely hydrophilic through a covering of protein cells in 20 min, and the character transformation indicated that cell attachment was not being prevented, even if the measured water contact angle was beyond 90°. The bactericidal capability of the moth-eye nanostructure was evaluated compared to smooth substrates, where a significant increase of the cell inactivation was observed on the processed surface for all tested bacteria. The topographical effect on the morphology of the HaCaT cells was also assessed, and when compared with the smooth substrate, there were no significant changes were detected in the SEM image, which verified the biocompatibility of moth-eye mimetic topography toward HaCaT cells.

The factors that determine the antibacterial capability and bactericidal efficiency of a nanostructured surface still remain ambiguous, so Ivanova et al. [59] executed an investigation into the role of nanopillar height toward clinically pathogenic bacteria. Deep ultraviolet (UV) immersion lithography and plasma etching were employed together to fabricate a highly ordered array of vertical silicon nanopillars of increasing height, equal diameter, and interpillar distance. The different heights of nanopillars were achieved by incrementally increasing the reactive ion etching time, which includes three dimensions (~220 nm, ~360 nm, and ~420 nm). The direct precise relationship between spatial geometry and resultant bactericidal efficiency could be explained by the isolation of a single geometrical parameter. Among the three dimensions, nanopillars 360 nm in height exhibited the highest degree of bactericidal activity, which inactivated ~95% *P. aeruginosa* and ~83% *S. aureus* cells. Instead of stable bunches of nanopillars, pillar deformation was induced during the cell adhesion process. The flexibility of the individual nanopillars was hypothesized to be responsible for the increasing energy storage when increasing the pillar height, but there is a critical point where it becomes energetically cheap to assemble an effectively continuous surface packed with nanopillar tips, which is pillar collapse. Therefore, mechanical energy stored substantially increased from a 220 to 360 nm pillar height, and the bactericidal effect can be partially compensated by irreversible interpillar adhesion when it reaches 460 nm in height.

### 4.2. Systematic Analysis on Biomimetic Basis and Derivates

The limited comprehensive studies in bactericidal properties of nanostructured surfaces are due to the arbitrary nanostructure patterns and manifold selected substratum material, and there is no definite theory to describe the bactericidal effect and cell rupture mechanism. As the pertinent researches into bactericidal surfaces escalates, the numerous collected experimental data and observed results could contribute to obtaining empirical routines beneath the various phenomena and determining the predominant factors in the topographical effect.

The main differences between contemporary antimicrobial surfaces regardless of natural surfaces or synthetic surfaces are the mechanical properties of the composed material and geometrical features of the nanostructures. In the beginning, Ivanova et al. [13] had negated the hypothesis about surface chemistry influence by certifying the almost identical bactericidal capability of the coated native surface, which precludes the influence factor from surface chemistry. The experiment also removed the influential factors from wettability, as the hydrophobic cicada wing surfaces were also saturated by the adhering bacteria.

The current synthetic nanostructures vary simultaneously in the radius and interspace distance due to a lack of control regarding the spatial geometry parameters. The recent work reported by Ivanova et al. [59] made alterations in height and kept other structural parameters constant to investigate the single parameter’s impact on bactericidal efficiency. The height of the nanopillars were manipulated at 220 nm, 360 nm, and 460 nm, respectively. The results demonstrated that the bactericidal efficiency reached the peak on the nanostructures at 360 nm instead of at the highest value of 460 nm. The increasing mechanical energy storage due to nanopillar deflection could be partially compensated for by irreversible interpillar adhesion. Therefore, there was an optimal value for nanostructure aspect-ratio, which was complementary to the previous extrapolation that the higher aspect ratio would contribute to better performance in antibacterial activities [56]. Wu et al. [72] prepared several nanoscale structures with different surface roughness, average pillar density, height, and interpillar distance by UV nano-replication technology, where the diameter is held constant for convenient analysis of the influence factors. The nanostructure with a medium pillar density (~40 pillars μm^−2^) could have a higher bactericidal activity compared to high pillar density (~70 pillars μm^−2^) and low pillar density (<20 pillars μm^−2^). The latter two nanostructures possessed a close degree of bactericidal efficiency to the smooth control sample. This revealed that stretching degree is highly dependent on the interaction pattern of the cell membranes on the nanopillars instead of extrapolation about the linear relationship between bactericidal efficiency and surface parameters. The height inhomogeneity of the nanopillar structure was discovered to effectively stimulate the stretching degree enhancement, which is also consistent with the previous theoretical model [8].

## 5. Prospects for the Development of Biomimetic Bactericidal Surfaces in Soft Contact Lenses

### 5.1. Bacterial Infection on SCL

SCL has been a common means of vision correction for almost half a century, since the release of the first commercial soft hydrogel lenses by Bausch and Lomb in the early 1970s [73]. Recent new contact lenses touting enhanced moisture retention properties indicates that a comfortable wearing experience is the primary customer selection factor, but the hygiene property of contact lenses persists as a considerable part in the design strategy. Customized SCL is commonly required for the specific optical performance control [74], but antibacterial SCL could intrigue customers who have a demand for high hygiene level.

Microbial keratitis (MK) [75], contact lens induced acute red eye (CLARE) [76], corneal infiltrative events (CIE) [77], and contact lens induced peripheral ulcers (CLPU) [78] are major types of serious bacterial infection, the ramifications of which are corneal damage, potentially leading to blindness, especially in the absence of adequate patient care and education. Bacterial adhesion to SCL is a major risk factor for microbial keratitis, and diminishment of bacterial adhesion is an efficient approach to prevent microbial keratitis. The main bacterial infection source of microorganisms attached on SCL includes hands [79], eyelids [80], lens care solutions [81], and storage cases [82]. Common pathogenic bacteria [83] involved in ophthalmology bacterial infection include *P. aeruginosa*, *S. aureus*, *S. marcescens*, and *Acanthamoeba* spp.

Current empirical disinfection strategies for SCL include killing the attached microorganisms in a chemical approach and altering the SCL wearing schedule, but both strategies have limited effect on the antibacterial activities. Since the invention of daily disposable SCL, the requirements of regular maintenance and sterilization have been abandoned, which was a vital cause of microbial contamination from storage and disinfection solution. Although the novel utilization mode of daily disposable SCL has been designated as a significant innovation in the development of SCL hygiene, Dart et al. [84] conducted a case-control study to assess the relative risks of microbial keratitis for different types for contact lens and wearing schedules, which demonstrated that there was no direct relationship between wearing mode and risk for contamination. Instead, the SCL design and composing biomaterial have a higher influence on the susceptibility to MK, so further research is required to validate the assumption due to limited sample size. For the chemical approaches, in vitro antimicrobial activities of melimine-coated contact lenses were observed on a rabbit model colonized by *P. aeruginosa* in the absence of corneal scratch [85]. The results manifested that the antimicrobial coat could reduce incidence of MK compared to the uncoated samples, but the progress of topical antibiotic development cannot keep pace with the rapid evolution of antibiotic resistance, which decreases the long-term prospects in commercial application. In recent years, the model concerning therapeutic contact lenses embedded with drugs or antimicrobial peptides has been proposed to control ophthalmic drug delivery, which improves the efficiency of drug delivery [76,77]. The method of drug release rate manipulation implicated a promising solution of storing some disinfection drugs in SCL to inactivate the attached microorganisms. The antibacterial properties of contact lenses containing metallic nanoparticles were assessed to validate the feasibility of the combination of bacterial contamination control and material composition modification [78,79]. The silver and copper impregnated hydrogel material successfully imparted an antibacterial capability sufficient to reduce the risk of bacterial infections, but only the polyvinyl alcohol (PVA) polymer containing copper was not cytotoxic, which indicates compensated action is required for eliminating cytotoxicity to human cells.

The efforts of adding antimicrobial peptides in a disinfection surfactant and storage case have achieved limited progress in infection reduction [71,72], hence, the introduction of a mechano-bactericidal surface to SCL surfaces may provide a potential feasible method to obviate bacterially-driven adverse events consistently.

### 5.2. Predisposing Factors of SCL-Induced Biofouling

Wearing SCL alters the state of the ocular surface, so the balance of corneal homeostasis is also disrupted, compromising the natural defenses that predispose patients to bacterial infections and sight-threatening complications. As a hydrophilic surface immersed in the interface between an aquatic environment and the atmosphere, SCL are not only a barrier between the corneal epithelium and oxygen, but also an optimal hotbed of bacterial incubation. In previous studies in SCL biofouling [16,67,68,70,73,74,80,81,82,83,84,85,86,87,88,89,90,91,92,93,94,95,96,97,98,99,100,101], the relationship between SCL physical properties and susceptibility to microbial adsorption have been discussed and analyzed to conceive specialized antibacterial strategies.

Surface roughness is a crucial geometrical parameter of surface characteristics, which is in direct contact with surrounding live organisms, and has a biological influence on the bacterial adhesion manner [96]. Higher surface roughness would increase the available active contact area for thermodynamic reactions, and surface irregularities like peaks and troughs serve as shelters for bacteria to promote the survival rate and avoid meeting unfavorable environmental factors [102]. After a long time or extended wearing, the surface roughness will generally increase due to dehydration and protein deposition as the extensive bacteria are prone to adhere to the SCL surface with increasing roughness [103].

The composed biomaterials of SCL are water-swollen, cross-linked, hydrophilic polymers, so polymerization of multiple monomers incorporate properties to form a biocompatible copolymer that provides a flexible approach to change the SCL characteristics [104]. The equilibrium water content (EWC) of SCL is the crucial parameter in the SCL design and manufacturing process, which has a direct relationship with the comfort degree and oxygen permeability (Dk) [73], where D is the material diffusivity and k is the material solubility. The several studies in EWC and bacterial adhesion depicted that bacterial adhesion increased inversely to the water content [103,105], but Miller et al. [106] showed that there was no stable correlation between EWC and adherence. The relationship between EWC and *Dk* is shown below [107]:

(1) $$\mathrm{EWC}=\frac{\mathrm{weight}\;\mathrm{of}\;\mathrm{water}\;\mathrm{in}\;\mathrm{polymer}}{\mathrm{total}\;\mathrm{weight}\;\mathrm{of}\;\mathrm{hydrated}\;\mathrm{polymer}}\times 100$$

(2) $$Dk=1.67e^{0.0397\mathrm{EWC}},$$

where e is the natural logarithm.

**Figure 4:**
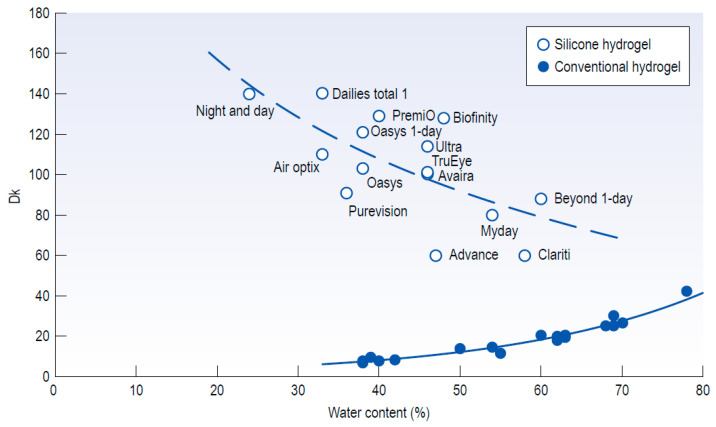
Relationship between *Dk* and EWC for conventional hydrogel and silicone hydrogel lenses, unit *Dk* (Barrer) = $10^{-11}\left({\mathrm{cm}}^{2}*\mathrm{ml}O_{2}\right)/\left(s*\mathrm{ml}*\mathrm{mm}Hg\right)$ [73].

SCL with sufficient oxygen permeability can maintain normal corneal metabolism and avoiding hypoxic, different composed biomaterials shown in Figure 4 present a contrary tendency in the relationship between *Dk* and EWC. The major difference between conventional hydrogel SCL and silicone hydrogel SCL is the chemical group composition, variety of chemical groups compose of the backbone of hydrogel which are used to attracting and binding water. The chemical groups containing silicon-oxygen bonds increase the oxygen permeability for silicone hydrogel SCL, because oxygen is more soluble in silicone rubber than that in water while oxygen is more soluble in water than that in polymethyl methacrylate (PMMA) [108].

Increasing oxygen permeability for a hydrogel is commonly achieved by simply adding EWC that exists at upper limit, where the highest oxygen transmissibility attained via EWC modification is theoretically far too deficient to meet the requirement of overnight wearing. To obtain the required *Dk*, the thickness of the SCL should be controlled within 0.06 mm, which is unfeasible for current manufacturing technology and stable fit to wear [107]. The dehydration rate of the anterior surface of hydrogel contact lenses is proportional to water content [109], so the dehydration behavior could lead to tear film stagnation and corneal dryness at the end of the day.

As vital surface characteristics of SCL, wettability has a direct effect on the interactions with tear film and biocompatibility in the ocular environment. Wettability of SCL is a measurement to describe the ability to support a continuous tear layer for corneal homeostasis and visual clarity, and can be divided into two categories including hydrophobicity and hydrophilicity. In the process of identifying the lens deposits and constituents on hydrophilic SCL, the hydrophilic property was discovered to reinforce the tear components adsorption like lipids, proteins, and mucins, which led to depreciated wearing comfort and visual clarity decline [110].

In a study on the role of hydrophobic forces, Michael et al. established that hydrophobic bacteria have the tendency to adhere to hydrophobic surfaces in the process of bacterial adhesion [111]. When executing the experiment on *P. aeruginosa* adhering to contact lenses, Fletcher et al. [105] found the role of hydrophobic O-side chains in lipopolysaccharide (LPS) molecules in adherence to contact lenses may increase the affinity of *P. aeruginosa* to specific charged molecules on the surface of the contact lenses. Despite the high oxygen permeability of silicone hydrogel contact lenses, the hydrophilicity of a hydrogel polymer incorporating silicone is minimized [108], and unworn silicone hydrogel contact lenses possess a higher susceptibility to *S. epidermidis* adhesion than conventional hydrogel contact lenses [112].

### 5.3. Nanostructure Fabrication on the Hydrogel Materials

Although hygiene issues are important in SCL design, SCL is a fundamental approach for vision correction with portability. Therefore, adding the proposed antimicrobial properties to SCL must retain the original viability and functionality, and the processed SCL ought to reserve all the prerequisite mechanical and optical properties to meet the requirement of wearing comfort and vision correction. The ideal refractive index for SCL is similar to that of the corneal (~1.37); the refractive index has an adverse linear relationship with EWC that is not applicable to silicone hydrogel lenses [73]. As described above, the EWC of SCL governs the *Dk* and dehydration rate, as the EWC of the hydrogel material is sensitive to outer environmental changes like temperature, pressure, and light. In the process of typical SCL manufacturing, there is a dry state for the inflexible monomer before immersion in saline solution [113], which is the suitable phase for nanostructure fabrication. After hydrating in saline, the anhydrous lens starts swallowing water and swells to the required dimensions and power, where the influence factors of swell behavior are temperature, pH, and tonicity [73]. To obtain the effective nanostructure at the required size, the dimension of the nanostructure fabricated on the anhydrous lens will be manipulated based on the swell factor of the biomaterial.

As above-mentioned, the nanostructure on the native insect wings was found to be damaged by cell movement where the Young’s modulus of nanostructure is much weaker than that of the substratum material. Due to the lack of relevant information about nanostructure fabrication on soft materials, the durability of the nanostructure on a hydrogel material needs further investigation. The modulus of the silicone hydrogel SCL was higher than the conventional hydrogel SCL [73], where a higher modulus possesses higher feasibility of industrial manufacturing and lower risk of nanostructure distortion. By comparing the material properties of contemporary conventional contact lenses and silicone contact lenses, silicone hydrogel could be a promising substratum material for nanoarchitecture construction on the SCL surface to relieve from bacterial contamination based on the contact-killing mechanism.

Alongside retaining the functionality of SCL, fabricating the nanostructure on a surface with a curvature is also one obstacle for industrial mass production. A nanostructure fabricated on a lens to obtain an anti-reflection coating through a combination of cast molding and the interfered femtosecond laser inscription technique provided a low-cost method [114]; nanoreplication techniques like nanoimprint lithography and soft lithography could develop a nanoscale complex nanostructure by pattern transferring [115], and the feasibility of these approaches to fabricate a nanostructure on anhydrous stage biomaterial requires further investigation.

Hydrogel is one conventional material in wet condition on which it is difficult to perform direct micro- or nanofabrication. Hydrogel applications in the medical field have gained popularity in recent years due to its high similarity to in vivo soft tissues as well as characteristics like high oxygen permeability and water-soluble metabolites that contribute to remarkable biocompatibility and adjustable physico-chemical properties [116]. Cast molding is a cost-effective technique for mass production in industrial manufacturing processes to fabricate a hydrogel-based structure with high precision using a damage-free demolding method and novel mold are proposed to achieve topological structures with diameters ranging from 500 nm to 100 μm [117].

As above-mentioned, another way to obtain a nanostructured surface for hydrogel materials is to create nanofeatures on top of the monomer in anhydrous status and achieve the exact dimension after water-swallowing. Considering the swell factor of the hydrogel materials, the initial size of the nanostructure should be controlled. Therefore, the precision requirement of processing techniques is higher than direct fabrication methods, and several approaches like nanoimprint lithography [67] or plasma etching [118] have been applied in nanostructure fabrication on polymeric surfaces that are sufficient for high-aspect-ratio structure fabrication within 100 nm.

Compared to rigid contact lenses, SCL has a larger edge area surrounding the optical zone. Fabricating a high-aspect-ratio nanostructure on the edge area could be a potential solution to reduce bacterial keratitis induced by bacterial infection, but the verification of biomimetic surface functionality on a soft hydrogel material requires further investigation.

## 6. Conclusions

Through previous studies on the bactericidal property of a biomimetic surface, the viability and feasibility of the antimicrobial model has been validated to effectively control the bacterial adhesion and biofilm formation. The threat from antibiotic-resistant germ evolution is expected to be limited by the further utilization of the mechano-ruptured mechanism in hygiene products as well as the risk of nosocomial infection. In the previous case, the bacterial incubation concentration is small and the adsorption mechanism requires further detailed investigation to provide a theoretical foundation for manufacturing. The durability of the functional nanostructure on the soft substratum is expected to differ from the hypothesized rigid model; the risk of structural distortion and deformation induced potential inner stress are needed to be supplementary. Quantification of the unique characteristics of surface morphology features and the design of a nanostructured surface contribute to providing a potential solution to impart bacterial resistance to various biomedical applications like soft contact lenses. It is also imperative that antimicrobial surfaces in real clinical cases be used judiciously to consider both biocompatibility and durability to minimize the risk of adverse effects due to intrinsic defects in design.

## Figures and Tables

**Table 1:** Pathogenic bacteria samples.

Bacteria	Size	Morphology	Source	Infections
Gram negative				
*Escherichia coli*	2 μm long, 0.25–1 μm diameter	Rods	Contaminated food, personal contact	Watery diarrhea, abdominal cramping, nausea, vomiting, urinary tract infection
*Pseudomonas aeruginosa*	1.5–3 μm long, 0.5–0.8 μm diameter	Rods	Water, soil	Dermatitis, cystic fibrosis, most bacterial cause of microbial keratitis in contact lenses wearers
*Pseudomonas fluorescens*	1–3 μm long, 0.5–0.7 μm diameter	Rods	Plants, soil, water surfaces	Blood transfusion-related septicemia, catheter-related bacteremia, peritonitis
*Klebsiella pneumoniae*	2 μm long, 0.5 μm diameter	Rods	Personal contact, indwelling catheters	Respiratory tract infections, urinary tract infections, endophthalmitis, skin and soft tissue infections, meningitis
Gram positive				
*Staphylococcus aureus*	0.6 μm cell diameter	Coccal	Nose, respiratory tract, direct personal contact	Bloodstream infections, endocarditis, osteomyelitis
*Bacillus subtilis*	4–10 μm long, 0.25–1 μm in diameter	Rods	Soil	Food contamination
*Enterococcus faecalis*	0.6–2 μm by 0.6–2.5 μm	Coccal	Gastrointestinal tract	Urinary tract infection, endocarditis, abdominal and pelvic infection, septicemia

**Table 2:** Naturally occurring bactericidal surfaces.

Natural Resource	Surface Features	Wettability	Bactericidal Efficacy	Lethality	Reference
Cicada wing (*Psaltoda claripennis*)	Nanopillar (200 nm height, diameter 100 nm at the base, diameter 60 nm at the cap, and spaced 170 nm apart from center to center)	Superhydrophobic Average water contact angle: 158.8° (147°–172°)	Individual cells were killed within approximately 3 min	*Pseudomonas aeruginosa*	[13]
Cicada wing (*Psaltoda claripennis*)	Nanopillar (200 nm height, base diameter 100 nm, cap diameter 60 nm, space 170 nm)	Hydrophobic Water contact angle: 158.8°	*P. aeruginosa*–$\left(6.14\pm 1.50\right)\times 10^{6}{\;\mathrm{cfu}\;\mathrm{cm}}^{-2}$ No remarkable effect on the viability of gram-positive cells	*Branhamella catarrhalis* *Escherichia coli * *Pseudomonas aeruginosa* *Pseudomonas fluorescens* *Planococcus maritimus*	[12]
Dog day annual cicada (*Tibicen tibicen*) Brood II periodical cicada (*Magicicada septendecim*)	Spherically capped cone (183 nm height, 104 nm base diameter, 57 nm cap diameter, spacing 175 nm) Hemisphere (83.5 nm height, 167 nm width, 252 nm spacing)	Hydrophobic Water contact angle: 132°, 80.1°	Dog day annual cicada–25% contamination comparing to control sample Brood II periodical cicada–54% contamination comparing to control sample	*Saccharomyces cerevisiae*	[9]
Cicada wing (*Megapomponia intermedia*, *Cryptotympana aguila*, *Ayuthia spectabile*)	Nanopillar (241 nm height, 165 nm pitch, 156 nm diameter, 9 nm spacing) Nanopillar (182 nm height, 187 pitch, 159 nm diameter, 28 nm spacing) Nanopillar (182 nm height, 251 nm pitch, 207 nm diameter, 44 nm spacing)	Hydrophobic Water contact angle: 135.5°, 113.2°, 95.65°	The bacterial live ratio for M. intermedia, C. aguila, A. spectabile, respectively is 0.222, 0.123 and 0.067	*P. fluorescens*	[7]
Dragonfly (*Diplacodes bipunctata*, *Hemianax papuensis*, *Austroaeschna multipunctata*)	Height 200–300 nm, top diameter 80 ± 20 nm, interpillar spacing 180 ± 30 nm	Hydrophobic Contact angle: ~(152°–162°)	13.0 × 10^4^ to 47 × 10^4^ cell killed per cm^2^ per min	*P. aeruginosa* *S. aureus* *B. subtilis* *B. subtilis spores*	[63]
Gecko skin (*Lucasium steindachneri*)	Length 2–4 μm Base thickness and spacing ~ 500 nm	Hydrophobic Contact angle: 150° ± 5°	88% *P. gingivalis* killed 66% *S. mutans* killed	*Porphyromonas gingivalis Streptococcus mutans*	[64]
Dragonfly wing (*Orthetrum villosovittatum*)	Height (short pillar 189 ± 67 nm, tall pillar 311 ± 52 nm) Pillar diameter (short pillar 37 ± 6 nm, tall pillar 57 ± 8 nm)	N/A	$2.33\times 10^{5}\pm \;5.83\times 10^{4}\;\mathrm{cells}{\;\min}^{-1}{\mathrm{cm}}^{-2}$	*Escherichia coli*	[8]
Damselfly (*Calopteryx haemorrhoidalis*)	Height 433.4 ± 71.2 nm Tip diameter 47.7 ± 11.1 nm Interspacing distance 116.1 ± 39.6 nm	Contact angle: 157.0° ± 4.9°	*P. aeruginosa* 97.9 ± 33.6% *S. aureus* 89.2 ± 36.0%	*P. aeruginosa* *S. aureus*	[65]
Dragonfly wing (*Austrothemis nigrescens*; *Trithemis annulata*)	*A. nigrescens* (height 307 ± 34 nm, diameter 45 ± 7 nm) *T. annulate* (height 292 ± 34 nm, diameter 45 ± 7 nm)	Contact angle (*A. nigrescens*: 162° ± 8°, *T. annulate*: 167° ± 6°)	Cell was ruptured within 3–5 min	Giant unilamellar vesicle	[10]

**Table 3:** Artificial biomimetic surface.

Substratum Material	Natural Templates	Fabrication Method	Geometrical Features	Wettability	Bactericidal Efficacy	Lethality	Reference
Silicon	Dragonfly	Reactive-ion beam etching	Height 500 nm	Hydrophilic Contact angle 80°	Killing rate *P. aeruginosa*: 4.3 × 10^5^ per cm^−2^min^−1^ *S. aureus:* 4.5 × 10^5^ per cm^−2^min^−1^ *B. subtilis:* 1.4 × 10^5^ per cm^−2^min^−1^	Gram-negative bacteria Gram-positive bacteria Spores	[62]
Silicon	Dragonfly	Deep reactive ion etching	Height 4 μm Diameter 220 nm	Hydrophobic Contact angle 154°	86% of *S. aureus* and 83% of *E. coli* were non-viable after 3 h incubation	Gram-positive bacteria Gram-negative bacteria Mammalian cell	[66]
PMMA	Cicada	Soft lithography	Height 210–300 nm Spacing 100–380 nm Width 70–215 nm	N/A	*E. coli:* 16–141% higher dead fraction than a flat film	Gram-negative bacteria	[67]
Silicon	Cicada	Metal assisted etching	Height 200 nm Pitch 200 nm Width 150 nm		*E. coli*: 24 h from 3.9 × 10^6^ CFU/mL to 1 CFU/mL	Gram-negative bacteria	[68]
Quartz	N/A	Nanosphere lithography	Height 300 nm Apex diameter 10 nm	Hydrophilic Contact angle ~ 0°	Kill ~38,000 *P. aeruginosa* and ~27,000 *E. coli* cm^−2^min^−1^	Gram-negative bacteria	[69]
PMMA	Moth-eye	Thermal polymer nanoimprint	Height 350 nm Width 80 nm Pitch 250 nm Aspect ratio 4.3	Hydrophobic 135 ± 4°	Percentage of non-viable bacteria are 55%, 45%, 30% for *S. aureus*, *E. coli*, and *P. aeruginosa* respectively	Gram-positive bacteria Gram-negative bacteria	[70]
Silicon	Cicada	Deep ultraviolet immersion lithography Plasma etching	Diameter 35 nm Periodicity 90 nm Increasing height 220, 360, 420 nm	N/A	360 nm-height 95 ± 5% *P. aeruginosa* and 83 ± 12% *S. aureus* cell death	Gram-negative bacteria Gram-positive bacteria	[59]
